# Pediatric psoriasis: Understanding pathological conditions and advances in treatment

**DOI:** 10.1111/1346-8138.17049

**Published:** 2023-12-17

**Authors:** Akimichi Morita, Hidehisa Saeki

**Affiliations:** ^1^ Department of Geriatric and Environmental Dermatology Nagoya City University Graduate School of Medical Sciences Nagoya Japan; ^2^ Department of Dermatology Nippon Medical School Hospital Tokyo Japan

**Keywords:** chronic disease, keratinocytes, psoriatic arthritis, pustular psoriasis, skin diseases

## Abstract

Psoriasis is a long‐lasting skin disease that primarily affects the skin, nails, and joints and is characterized by inflammation. Genetic factors contribute to its development and environmental triggers can worsen symptoms. Pathologically, psoriasis is characterized by uncontrolled keratinocyte proliferation and abnormal differentiation, and histological features include acanthosis with inflammatory cell infiltration and neovascularization. Psoriasis often starts in childhood, with about one‐third of cases beginning during this time. Its prevalence steadily increases from the ages of 1 to 18 years in a linear fashion. Young people with psoriasis often require treatment throughout their childhood and adolescence, and into adulthood. However, prolonged treatment may increase the risk of complications and adverse events, so it is important to adopt an effective treatment approach that minimizes this risk. In addition, psoriasis is often associated with various comorbidities that may place a great burden on the physical and mental health of the children beyond those due to psoriasis itself. To ensure good long‐term health outcomes, individuals with psoriasis should undergo regular screening. Treatment should be provided not only for skin lesions, but also for any comorbidities; however, currently there is not enough evidence on the treatment of pediatric psoriasis and no globally agreed‐on guidelines exist for treating psoriasis in children. This article describes the etiology, clinical symptoms, and disease burden of pediatric psoriasis, the pathological conditions and diagnosis of plaque psoriasis, psoriatic arthritis, and generalized pustular psoriasis, and the available treatments for these conditions in Japan.

## 1 INTRODUCTION

Psoriasis is a chronic inflammatory skin disease that is exacerbated by environmental factors on a background of genetic predisposition; lesions primarily affect the skin, nails, and joints.^1^ Pathologically, psoriasis is characterized by uncontrolled keratinocyte proliferation and dysfunctional differentiation, with histological features that include acanthosis with inflammatory cell infiltration and neovascularization.^1^ A study in Germany reported that the prevalence of psoriasis in children is 0.71%.^2^ Psoriasis begins in childhood in almost one‐third of cases,^3^ and in the United States, the annual incidence of pediatric psoriasis was reported to be 40.8 per 100 000.^4^ The German study showed that the prevalence of psoriasis in children increases in an approximately linear manner from 0.12% to 1.24% from the age of less than 1 year to 18 years.^2^ In a Japanese Society for Psoriasis Research survey of 15 287 psoriasis patients (from 2013 to 2018), pediatric psoriasis occurred at 0 to 9 years in 266 patients (2.0%) and at 10 to 19 years in 1103 patients (8.1%).^5^ International studies have shown that pediatric psoriasis is more common in girls than in boys, but the difference is not always significant, and that in adults, psoriasis appears to be slightly but nonsignificantly more common in women than in men, although one study found a significantly higher incidence in men.^4^ However, the situation among adults in Japan is different in that the ratio of men to women is 2:1.^5^

Children with psoriasis require treatment until adulthood, and prolonged treatment may increase the risk of complications and adverse events, therefore it is crucial to adopt an effective treatment approach that reduces this risk. In addition, patients with psoriasis are likely to have various comorbidities.^6^ The prevalence of comorbidities in patients with psoriasis younger than 20 years old (14.4%) is nearly twice that of children without psoriasis (7.2%), including those with hyperlipidemia, hypertension, diabetes mellitus, rheumatoid arthritis, and Crohn's disease.^7^ Additionally, long‐term comorbidities associated with psoriasis may place a great burden on the physical and mental health of children with psoriasis beyond the effects of psoriasis itself. Therefore, in consideration of the long‐lasting impact of psoriasis on health, patients must be screened periodically and receive treatment not only for their skin lesions but also for comorbidities. However, there is insufficient evidence regarding the medical treatment of pediatric psoriasis and no internationally harmonized guidelines have been developed to date.^8^ National guidelines for the treatment of pediatric psoriasis include the 2019 Joint American Academy of Dermatology–National Psoriasis Foundation guidelines of care for the management and treatment of psoriasis in pediatric patients,^9^ the 2019 guidelines for the treatment of psoriasis in children and adolescents, jointly prepared by German dermatologists, pediatric dermatologists, and pediatric rheumatologists,^10^, ^11^ and the 2022 consensus on the management of pediatric psoriasis prepared by Italian dermatologists.^12^ In Japan, guidelines for psoriasis include the 2014 guidelines for the management and treatment of generalized pustular psoriasis (GPP),^13^ the 2019 guidelines for the treatment of psoriatic arthritis (PsA),^14^ the 2016 guidelines for phototherapy in psoriasis,^15^ and the 2022 guidance for the use of biologics for psoriasis.^16^ There are currently no specific guidelines for treating pediatric psoriasis. Furthermore, the safety of some drugs has not been established for use in children with this condition, and fewer treatment options are available than for adults.^17^ This review provides information on pediatric psoriasis, including its causes, symptoms, and health impact. It also covers the diagnosis and treatment of plaque psoriasis, PsA, and pustular psoriasis, with a focus on GPP, and provides information on the available treatments in Japan for these conditions.

## 2 ETIOLOGY AND CLINICAL SYMPTOMS OF PEDIATRIC PSORIASIS

The onset of pediatric psoriasis involves various factors, including the stress associated with starting school, separation from parents, mechanical trauma, and infection.^7^ Immunological factors include interactions between various kinds of immune cells, such as T‐helper cell 1 (Th1), Th17, Th22, mast cells, and neutrophils, and abnormal expression of cytokines.^6^ One review reported that the onset of psoriasis primarily involves the cytokines such as interferon (IFN)‐γ, tumor necrosis factor (TNF)‐α, and interleukin (IL)‐17, IL‐22, and IL‐23 and that an imbalance between effector T cells, such as Th‐17, and regulatory T cells plays a role in the etiology of psoriasis.^7^, ^18^ Moreover, the onset of psoriasis may involve LL‐37, an antimicrobial peptide that activates plasmacytoid dendritic cells and keratinocytes, leading to the production of various inflammatory cytokines.^19^ IL‐36 is also considered to be involved in the onset of psoriasis by stimulating keratinocytes to produce inflammatory cytokines such as TNF‐α and IL‐17C.^20^ In psoriasis, inflammation repeatedly occurs at the same sites, apparently because of the involvement of local immunological memory associated with tissue‐resident memory T cells (TRM), suggesting that re‐exposure to antigens in the presence of TRM and the production of inflammatory cytokines by TRM may precipitate the inflammatory response.^21^ Some genes involved in the onset of psoriasis have also been identified, and positivity for the human leukocyte antigen (HLA) alleles *HLA‐Cw6* and *HLA‐Cw1* was associated with high risk of developing psoriasis.^22^

The main types of psoriasis are plaque psoriasis, which exhibits only skin symptoms; PsA, in which skin symptoms are complicated by joint symptoms; GPP, which causes general symptoms in which skin rash coexists with pustules; guttate psoriasis, which features a small rash; and erythrodermic psoriasis, which features systemic lesions.^23^ Other types include scalp psoriasis, anogenital psoriasis, and nail psoriasis, which exhibit local lesions (Table 1). Tissue biopsies may be prepared if it is difficult to discriminate psoriasis from other diseases, but it is common to make a clinical diagnosis on the basis of the characteristic shape and distribution of the skin lesions.

**TABLE 1:** Characteristics of the different types of pediatric psoriasis.

Plaque psoriasis
Most common clinical type; accounts for 41% or more of psoriasis cases in children (aged ≥2 to <13 years) and adolescents (aged ≥13 years)^24^ Chronic plaque psoriasis occurs in up to 75% of children with psoriasis^3^ Characterized by a well‐defined erythematous plaque covered with micaceous scales^25^, ^26^
Guttate psoriasis
Accounts for 15%–30% of cases of pediatric psoriasis^10^, ^25^ More common in children than in adults^27^ Characterized by the rapid onset of guttate papular lesions associated with infection^10^, ^25^
Diaper psoriasis
Most common type; accounts for 37% of cases in infants with psoriasis^25^ Exhibits a well‐defined florid and occasionally eroded plaque^25^
Inverse psoriasis
Second most common type; accounts for 22.2% of cases in infants with psoriasis^25^ Causes lesions in the skin folds (in the armpits and groin) more often than in adults and in the anogenital area because of particular rubbing^10^
Erythrodermic psoriasis
Very rare in children but potentially life‐threatening^3^ Characterized by erythema covering more than 90% of the body surface area, as well as scales^3^
Pustular psoriasis
Occurs in 1.0%–5.4% of children with psoriasis^3^ More common in adults than in children^3^ Characterized by localized or systemic superficial aseptic pustules^3^ Acute generalized pustular psoriasis and annular pustular psoriasis frequently occur in childhood^3^
Facial psoriasis
Only occurs in 4%–5% of children^28^ Erythema, scales, and plaque occur on the skin around the brow, nasolabial fold, mouth, or other facial regions^1^
Juvenile psoriatic arthritis
Occurs in 1%–10% of children with psoriasis^3^ Peak age of onset is 9–12 years^3^ Symptoms include swollen joints, sensation of pressure, pain when moving, feeling feverish, and motor impairment^10^
Nail psoriasis
Occurs in approximately 40% of children with severe psoriasis^25^ Less common in children than in adults^25^ Characterized by yellow subungual plaques (oil spots) and pitting in the nail plate^10^
Scalp psoriasis
Common (20%–50%) initial lesion site is the scalp^29^ Characterized by well‐demarcated erythematous plaques with superimposed silvery scales^25^

Other clinical features of psoriasis include the Auspitz sign, which is an occurrence of punctuated bleeding spots when the scales are scraped off, and the Koebner phenomenon, which is the occurrence of lesions in areas of trauma.^3^ Some of the symptoms of pediatric psoriasis are similar to those of adult psoriasis; however, pediatric psoriasis shows a predilection for involvement of the face and anogenital areas, and a higher prevalence of neonatal napkin/diaper psoriasis and guttate psoriasis compared with adults (Table 1).^7^ In children, the disease is more pruritic and the lesions are thinner, softer, and less scaly (because there is less infiltration).^29^ The fact that lesions show little thickening or scaling in pediatric psoriasis may lead to missed or delayed diagnosis.

The diseases that need to be differentiated from pediatric psoriasis are listed in Table 2. The symptoms of pediatric psoriasis may not be identical to those of adult psoriasis because of differences in pathological development, although in pediatric psoriasis, this process has not been sufficiently elucidated. A recent study revealed significant differences in TNF‐α and IL‐17 expression in skin lesions between pediatric and adult psoriasis patients,^30^ and another study showed significant differences in IL‐17 and IL‐22 expression in skin lesions in children with psoriasis compared with healthy children and adults with the disease.^7^

**TABLE 2:** Possible differential diseases for each type of psoriasis^27^.

Type of psoriasis	Differential diseases
Plaque psoriasis	Seborrheic dermatitis, nummular eczema, pityriasis rubra pilaris, body tinea
Guttate psoriasis	Drug eruption with erythematous papules, pityriasis rosea Gibert, pityriasis rubra pilaris, pityriasis lichenoides, lichen planus, body tinea, secondary syphilis
Pustular psoriasis (generalized)	Acute generalized exanthematous pustulosis, superinfected dermatitis, body tinea, Sweet syndrome
Pustular psoriasis (localized)	Inflamed nail head (infection), contact dermatitis with infection, dyshidrotic eczema with infection, Sweet syndrome, tinea
Scalp psoriasis	Tinea capitis, atopic dermatitis, seborrheic dermatitis
Anogenital psoriasis	Allergic contact dermatitis, lichen planus, lichen sclerosus, lichen simplex, zinc deficiency, candidiasis
Nail psoriasis	Nail tinea, body tinea, alopecia areata, nail deformity, lichen planus

Dermoscopy can be useful for diagnosing pediatric psoriasis. In this non‐invasive procedure, the skin is examined with a skin surface microscope. Diagnosis of pediatric psoriasis should be based on clinical evidence, but skin biopsies are often discouraged in pediatric patients, so dermoscopy can aid in diagnosis.

## 3 BURDEN OF PEDIATRIC PSORIASIS

Pediatric psoriasis places a great burden on children and their families. Children with psoriasis are likely to feel ashamed of their skin lesions and to experience loss of self‐confidence, anxiety, and social isolation.^12^ Furthermore, pruritus associated with psoriasis is a great cause of stress that disturbs daily living and sleep.^31^ Pediatric patients with moderate‐to‐severe plaque psoriasis demonstrated significantly impaired health‐related quality of life (QOL) in relation to physical, emotional, social, and school functioning compared with healthy children,^32^ and pediatric psoriasis was associated with significantly worse QOL than vitiligo, another skin disease.^33^ Children with psoriasis are at approximately 20%–30% higher risk of developing psychiatric disorders such as depression and anxiety than children without any psoriasis diagnosis.^34^ Anxiety or depression may stem from experiences of shame, behavior avoidance, bullying, decreased self‐confidence, and social isolation caused by psoriasis.^31^ A study that used the Family Dermatology Life Quality Index to evaluate the QOL of the parents of children with skin diseases showed that pediatric psoriasis places a substantial burden on parents, mainly due to the associated routine household expenditure, the time required to care for the child's skin, and the mental stress related to anxiety, depression, and other people's reactions to the child's disease.^35^ It was also reported that the impact of other people's reactions to the child's disease was greater in the parents of girls than in those of boys.^35^ In addition, both anxiety and depression were observed in 36% of the caregivers of children with psoriasis.^36^ Thus, psoriasis can significantly impact the QOL of not only the children but also their parents.

## 4 PLAQUE PSORIASIS

### 4.1 Epidemiology

Bonigen et al. reported that plaque psoriasis was found in 25.9% of infants under 2 years of age and in more than 41% of children and adolescents (aged ≥2 to <18 years old) and that it was the most frequent clinical type of psoriasis seen in children and adolescents.^24^ In addition, one article reported that approximately 70% of children with psoriasis present with chronic plaque psoriasis.^37^

### 4.2 Pathological conditions and symptoms

Plaque psoriasis is characterized by a well‐defined erythematous plaque covered with silver scales,^25^ with the Auspitz sign or Koebner phenomenon. Plaque psoriasis lesions are smaller, thinner, and less numerous in children than in adults, and children have fewer scaly lesions. Plaque psoriasis typically affects areas such as the scalp, forehead, face, postauricular region, periumbilical region, buttocks, and diaper contact area, and infections are important inducers of the disease.^26^

### 4.3 Diagnosis

Because the scales in pediatric plaque psoriasis are thin, scraping them causes punctate blood spots (Auspitz sign). Other diagnostic characteristics include lesions at sites of trauma (Koebner phenomenon) and residual pigmentation after a lesion has healed. The histological features of plaque psoriasis include parakeratosis, loss of the granular cell layer, elongation of the reticular ridges, neutrophilic aggregates within the epidermis (Munro's microabscesses), dilated blood vessels in the dermis, and perivascular lymphocytic infiltrates. Biopsy can help to establish the diagnosis of plaque psoriasis in children with atypical symptoms. However, because its diagnosis is usually based on morphology and distribution, biopsy is virtually never performed, especially not in children.^3^ Several skin conditions may be misdiagnosed as plaque psoriasis, including seborrheic dermatitis, nummular eczema, pityriasis rubra pilaris, and body tinea, so differential diagnosis is important to ensure proper treatment (Table 2).

### 4.4 Treatment

The treatment of plaque psoriasis is broadly divided into topical therapy, phototherapy, oral therapy, and biotherapy. Although the efficacy of topical steroids in children has not yet been adequately studied in clinical trials, these agents are widely used in clinical practice for the treatment of pediatric psoriasis.^9^ In Japan, no clinical studies have been performed on vitamin D3 preparations or topical combination products in children. For patients with an insufficient response to topical therapy, phototherapy is an option, and narrow‐band ultraviolet B is considered first‐line phototherapy because of its convenience.^15^ According to Japanese guidelines, phototherapy can be used in children, but the size of the treatment area and number of treatments should be kept to a minimum.^15^ Combining phototherapy and ciclosporin (note that cyclosporin is spelled “ciclosporin” in Japan) to treat psoriasis is not recommended because combination treatment can increase the chance of developing skin cancer.^15^ Because most cases of pediatric psoriasis are mild to moderate in severity, topical therapy is the most extensively used approach, whereas phototherapy and systemic therapy are limited to severe or refractory cases, PsA, or patients with decreased QOL.^26^ Oral preparations include vitamin A analogs, phosphodiesterase 4 (PDE4) inhibitors, and immunosuppressants (Table 3). Both etretinate, a vitamin A analog, and ciclosporin, an immunosuppressant, are indicated in children.^38^, ^40^ The package insert of etretinate states that, because of the risks of hyperostosis and premature fusion of epiphyses, infants and children should be observed closely.^38^ The package insert of ciclosporin states that children should be carefully selected for treatment and, if treated, their condition should be carefully monitored.^40^ Methotrexate, an antirheumatic drug, is indicated for the treatment of plaque psoriasis with an insufficient response to topical therapy, PsA, pustular psoriasis, and erythrodermic psoriasis, but it should be administered carefully to children.^41^ In Japan, no clinical studies in children with psoriasis have been performed for apremilast, a PDE4 inhibitor,^39^ upadacitinib, a Janus kinase inhibitor,^42^ or deucravacitinib, a tyrosine kinase 2 inhibitor.^43^

**TABLE 3:** Oral preparations for the treatment of psoriasis in Japan.

Type	Product name, reference	Generic name	Indication for psoriasis
Vitamin A analog	TIGASON^a^ ^,^ ^38^	Etretinate	Psoriasis group (plaque psoriasis, pustular psoriasis, erythrodermic psoriasis, psoriatic arthritis)
PDE4 inhibitor	Otezla^39^	Apremilast	Plaque psoriasis with insufficient response to topical therapy, psoriatic arthritis
Immunosuppressant	Neoral^40^	Ciclosporin	Plaque psoriasis, pustular psoriasis, erythrodermic psoriasis, and psoriatic arthritis
Antirheumatic drug	RHEUMATREX^41^	Methotrexate	Plaque psoriasis with insufficient response to topical therapy, psoriatic arthritis, pustular psoriasis, and erythrodermic psoriasis
Janus kinase inhibitor	RINVOQ^42^	Upadacitinib hydrate	Psoriatic arthritis
Tyrosine kinase 2 inhibitor	Sotyktu^43^	Deucravacitinib	Plaque psoriasis, pustular psoriasis, and erythrodermic psoriasis

Abbreviation: PDE4, phosphodiesterase 4.

^a^ The “Dosage and Administration” section of the package insert includes information on use in infants and children.

Systemic therapies are commonly considered if the lesion covers 10% or more of the body surface area, the Psoriasis Area and Severity Index (PASI) score is greater than or equal to 10, or the Dermatology Life Quality Index (DLQI) score is greater than or equal to 10.^16^ Biologics may be considered if the response to existing systemic therapies is insufficient.^16^ International and Japanese clinical studies of various biologics in adults showed high efficacy of biologics, with an improvement of 75% or more in the PASI score (PASI‐75) in 59%–95% of patients, improvement of 90% or more (PASI‐90) in 33%–92% of patients, and improvement of 100% (PASI‐100) in 10%–68% of patients.^16^ The available biologics are listed in Table 4. In children aged 6 years or older, psoriasis can currently be treated only with secukinumab because it is the only biologic approved for this use in Japan (as of December 2022).^16^ In these children, secukinumab should be subcutaneously administered every 4 weeks at a dose of 75 mg for children weighing less than 50 kg and at a dose of 150 or 300 mg for those weighing 50 kg or more.^47^ In a clinical study of secukinumab in children aged 6 years or older, PASI‐75 and PASI‐90 were achieved in 80% and 69%–71% of patients, respectively.^47^ Currently, there are no established criteria for selecting biologics for treatment of plaque psoriasis.

**TABLE 4:** Biologics for the treatment of psoriasis in Japan.

Product name, reference	Generic name	Year approved in Japan (for psoriasis)	Administration route	Target	Structure	Indication for psoriasis			
						Plaque psoriasis	Psoriatic arthritis	Pustular psoriasis	Erythrodermic psoriasis
REMICADE^44^	Infliximab	2010	Intravenous	TNF‐α	Chimeric mAb	〇	〇	〇	〇
HUMIRA^45^	Adalimumab	2010	Subcutaneous	TNF‐α	Human mAb	〇	〇	〇	
Stelara^46^	Ustekinumab	2011	Subcutaneous	IL‐12/23	Human mAb	〇	〇		
Cosentyx^a^ ^,^ ^47^	Secukinumab	2014	Subcutaneous	IL‐17A	Human mAb	〇	〇	〇	
Taltz^48^	Ixekizumab	2016	Subcutaneous	IL‐17A	Humanized mAb	〇	〇	〇	〇
LUMICEF^49^	Brodalumab	2016	Subcutaneous	IL‐17RA	Human mAb	〇	〇	〇	〇
Tremfya^50^	Guselkumab	2018	Subcutaneous	IL‐23	Human mAb	〇	〇	〇	〇
Skyrizi^51^	Risankizumab	2019	Subcutaneous	IL‐23	Humanized mAb	〇	〇	〇	〇
Cimzia^52^	Certolizumab pegol	2019	Subcutaneous	TNF‐α	PEGylated humanized mAb	〇	〇	〇	〇
ILUMYA^53^	Tildrakizumab	2020	Subcutaneous	IL‐23	Humanized mAb	〇			
Bimzelx^54^	Bimekizumab	2022	Subcutaneous	IL‐17A/F	Humanized mAb	〇		〇	〇
Spevigo^55^	Spesolimab	2022	Intravenous	IL‐36	Humanized mAb			〇	

Abbreviations: IL, interleukin; mAb, monoclonal antibody; PEG, polyethylene glycol; R, receptor; TNF, tumor necrosis factor.

^a^ The “Dosage and Administration” section of the package insert includes information on use in children aged 6 years or older.

## 5 PSORIATIC ARTHRITIS

### 5.1 Definition and epidemiology

PsA in patients younger than 16 years of age is referred to as juvenile PsA (JPsA).^14^ Chronic arthritis of unknown cause, including PsA, in patients younger than 16 years old is called juvenile idiopathic arthritis (JIA).^14^ JPsA is a type of juvenile spondyloarthritis with inflammation of trunk joints and peripheral joints, including hands and fingers, and is a type of JIA.^56^ There are no clear data on the prevalence of JPsA in Japan. According to a survey of pediatric rheumatism clinics in Japan, JPsA accounts for approximately 0.6% of JIA cases.^14^ Akioka reported that JPsA accounts for 14% of JIA cases.^57^ Assuming JPsA accounts for approximately 0.6% of JIA cases and the prevalence of JIA is approximately 1 out of 10 000 children in Japan, the prevalence of JPsA is estimated to be 0.00006%.^14^ In addition, in children with psoriasis, the incidence of JPsA was estimated to be 0.086% in Japan, lower than that in Europe and America,^14^ suggesting that JPsA is classified as another type of JIA because of difficulties in diagnosing pediatric psoriasis.^14^ One review reported that the onset of JPsA peaks between 9 and 12 years of age,^3^ and a study of 27 patients with JPsA in Japan reported that the mean age of onset was 11.2 years for psoriasis and 12.2 years for arthritis.^58^

### 5.2 Pathological conditions and symptoms

Two distinct age‐based populations have been identified among children with JPsA: one population comprises children younger than 5 years (median age 1.8 years) and the other comprises children aged 5 years and older and adolescents (median age 10 years).^59^ In the younger population, dactylitis and small joint involvement are more common and tests for antinuclear antibodies are more likely to be positive. Furthermore, although the disease usually remains oligoarticular, in this population more cases become polyarticular. By comparison, JPsA that develops in children aged 5 years and older and adolescents exhibits enthesitis and axial joint involvement and continues to show oligoarticular involvement.^59^ PsA, i.e., psoriasis with joint symptoms, involves joint deformity and destruction.^14^ Because of difficulties in diagnosing and classifying PsA in children, its prevalence ranges from 1% to 10% in children with psoriasis.^3^ Enthesitis and dactylitis occur in 40%–50% of patients with PsA.^60^ Joint lesions show a wide variety of severities and clinical courses and may occur in various joints.^61^ In patients with PsA, rash in psoriasis may be preceded by arthritis, or vice versa. Ogdie et al. reported that arthritis followed the diagnosis of psoriasis in 72.4% of patients, arthritis preceded psoriasis in 10.8%, and both occurred almost simultaneously in 16.8%.^62^ PsA is associated with many comorbidities. Inflammation‐related nail abnormalities such as nail pitting are observed in 50%–80% of cases and may be complicated by uveitis.^61^ As with plaque psoriasis, cytokines such as IL‐23, IL‐17A, IL‐22, and TNF‐α are considered to be involved in the onset of PsA.^63^

### 5.3 Diagnosis

The International League of Associations for Rheumatology classifies JIA into seven types. According to this classification, PsA falls under either (1) arthritis with psoriasis or (2) a condition with two or more of (A) dactylitis, (B) nail deformities (nail pitting, onycholysis, etc.), and (C) a parent or sibling with psoriasis. However, cases that meet one or more of the following conditions are considered unclassified arthritis rather than PsA: (1) arthritis in an *HLA‐B27*‐positive boy with onset after his sixth birthday; (2) ankylosing spondylitis, enthesitis‐related arthritis, sacroiliitis with inflammatory bowel disease, Reiter syndrome, or acute anterior uveitis in the patient or a first‐degree relative; (3) the presence of rheumatoid factor on at least two occasions at least 3 months apart; and (4) the presence of systemic JIA.^14^

### 5.4 Treatment

Japanese guidelines for the clinical practice of PsA have been developed primarily for adult patients, and no detailed guidelines have been published for the treatment of PsA in children. The Group for Research and Assessment of Psoriasis and Psoriatic Arthritis, which mainly covers America and Europe, created treatment recommendations by evaluating systematic reviews and through consensus, and gave a recommendation level of A for phototherapy, methotrexate, fumarate, TNF inhibitors, and ciclosporin as treatments for skin lesions in PsA.^64^ The treatment of PsA aims to reduce joint symptoms, improve QOL, and prevent joint deformity and destruction, and mainly consists of medical therapy to prevent the progression of joint symptoms.^14^

The treatment of joint symptoms starts with non‐steroidal anti‐inflammatory drugs (NSAIDs), of which only ibuprofen and naproxen are indicated in children.^14^ Agents indicated for the treatment of PsA are listed in Table 5. In addition to steroids (injectable and oral), other drugs indicated in children are etretinate,^38^ methotrexate,^41^ and ciclosporin^40^; however, careful patient selection and close observation are required for these three drugs. In the case of nonresponse to NSAIDs, patients should be switched to methotrexate, a conventional antirheumatic drug.^14^ Biologics should be considered for patients with no response or intolerance to methotrexate.^14^ TNF inhibitors are the first‐choice biologics based on the evidence for psoriasis and arthritis and are now available for use in Japan.^14^, ^44^, ^45^, ^52^ In addition, secukinumab,^47^ an anti‐IL‐17A monoclonal antibody, is indicated in children with PsA.^44^, ^45^, ^46^, ^48^, ^49^, ^50^, ^51^, ^52^ Rash in psoriasis should be treated as described for plaque psoriasis.

**TABLE 5:** Agents indicated for the treatment of psoriatic arthritis in Japan.

Type	Product name, reference	Generic name
Conventional antirheumatic drug	RHEUMATREX^41^	Methotrexate
Steroids	Various products	
Immunosuppressant	Neoral^40^	Ciclosporin
PDE4 inhibitor	Otezla^39^	Apremilast
Janus kinase inhibitor	RINVOQ^42^	Upadacitinib hydrate
Vitamin A analog	TIGASON^a^ ^,^ ^38^	Etretinate
Biologics		
TNF‐α inhibitors	REMICADE^44^	Infliximab
	HUMIRA^45^	Adalimumab
	Cimzia^52^	Certolizumab pegol
IL‐12/23p40 inhibitor	Stelara^46^	Ustekinumab
IL‐23p19 inhibitors	Tremfya^50^	Guselkumab
	Skyrizi^51^	Risankizumab
IL‐17 inhibitors	Cosentyx^b^ ^,^ ^47^	Secukinumab
	Taltz^48^	Ixekizumab
	LUMICEF^49^	Brodalumab

Abbreviations: IL, interleukin; PDE4, phosphodiesterase 4; TNF, tumor necrosis factor.

^a^ The “Dosage and Administration” section of the package insert includes information on use in infants and children.

^b^ The “Dosage and Administration” section of the package insert includes information on use in children aged 6 years or older.

## 6 GENERALIZED PUSTULAR PSORIASIS

### 6.1 Epidemiology

GPP is characterized by localized or systemic superficial aseptic pustules and develops in 1.0%–5.4% of children with psoriasis.^3^ Its prevalence in Japan is 7.46 out of 1 million people,^65^ peaking in childhood and the 30s, and is slightly higher in women.^13^ Pediatric GPP is rare, and the most common types appear to be the acute von Zumbusch type of typical GPP and annular pustular psoriasis.^3^, ^66^, ^67^

### 6.2 Pathological conditions and symptoms

Patients with GPP develop a systemic skin rash and multiple aseptic pustules, as well as rapid fever.^13^ GPP recurs repeatedly and, during its course, patients may exhibit systemic inflammatory response syndrome, mucosal symptoms, and arthritis, among other symptoms.^13^ GPP is sometimes preceded by plaque psoriasis.^65^ Depending on the disease course and clinical presentation, it is classified into (1) von Zumbusch type, (2) GPP preceded by plaque psoriasis, (3) annular pustular psoriasis, and (4) GPP in pregnancy, previously named impetigo herpetiformis.^68^ Patients with the von Zumbusch type, the most severe form of GPP, exhibit noninfective systemic eruption and systemic symptoms such as fever, fatigue, and nausea.^69^ Patients with annular pustular psoriasis show mild to no systemic symptoms and localized noninfective annular erythematous skin eruption.^69^ GPP in pregnancy is characterized by systemic noninfective erythematous skin eruptions.^69^

Cytokines, especially IL‐36, are considered to be greatly involved in the etiology of GPP, which is strongly linked to gene mutations that affect IL‐36, particularly in the genes *IL36RN*, *CARD14*, and *AP1S3*.^69^ *IL36RN* encodes the IL‐36 receptor antagonist, which controls the effects of IL‐36 in the production of inflammatory cytokines, including IL‐8.^13^, ^69^ Mutations in *IL36RN* enhance the inflammatory response caused by IL‐36.^69^ In a population of 64 patients with GPP, which included patients without mutations, one study found that the proportion of patients with the homozygous c.115+6T>C variant was 32.8%, the proportion with both heterozygous and homozygous c.115+6T>C variants was 45.3%, and the proportion with both heterozygous and homozygous c.227C>T variants was 17.2%.^70^ *IL36RN* variants are found in approximately 24% of patients with GPP.^69^ *CARD14* encodes the proteins involved in apoptosis and the control of the nuclear factor‐kappa B (NF‐κB) pathway. c.2458C>T is the most frequent mutation, and other mutations include c.1641C>T and c.1753G>A.^70^ Gain‐of‐function variants in *CARD14* appear to promote the inflammatory skin response via activation of the NF‐κB pathway and enhanced transcription of IL‐36, and occur in up to 21% of patients with GPP and concomitant plaque psoriasis.^69^, ^70^ *AP1S3* mutations include p.Phe4Cys and p.Arg33Trp.^71^ *AP1S3* mutations in conjunction with *IL36RN* changes are considered to increase the impact of *IL36RN* mutations and enhance the inflammatory response via IL‐36 overexpression.^71^

### 6.3 Diagnosis

To confirm a diagnosis of GPP, patients must exhibit all four of the following primary characteristics: (1) systemic symptoms, such as fever and fatigue; (2) systemic or extensive flush accompanied by multiple sterile pustules that sometimes merge to form lakes of pus; (3) neutrophilic subcorneal pustules histopathologically characterized by Kogoj's spongiform pustules; and (4) repeated occurrence of these clinical and histological features.^13^ GPP is suspected in patients with (2) and (3).^13^ Patients with the following can be excluded from initial onset of GPP: (1) in principle, clear cases of psoriasis vulgaris with transient pustule formation after the application of corticosteroids; (2) in general, the circinate annular form because of mild systemic symptoms; and (3) cases where subcorneal pustular dermatosis or pustular drug eruptions (including acute generalized exanthematous pustulosis) can be diagnosed after careful observation for a certain period of time.^13^

### 6.4 Treatment

GPP is a rare and life‐threatening systemic inflammatory disease. Because of its rarity, obtaining sufficient evidence‐based treatment guidelines can be challenging, meaning that, in some cases, drugs may have to be used that do not have well‐established safety profiles in children.^13^ To avoid cardiovascular failure, which is often fatal, systemic management and drug therapy are essential components of acute GPP treatment.^13^ According to Japanese GPP treatment guidelines, ciclosporin, an immunosuppressant, should be the first‐line drug in children with GPP, and etretinate or systemic steroid therapy is recommended for patients unresponsive to ciclosporin.^13^ TNF‐α inhibitors should be considered if those treatments are ineffective, for patients with severe joint symptoms, or if a rapid treatment response is needed.^13^ TNF‐α inhibitors, for which there is limited clinical experience in the treatment of pediatric GPP, might be considered in patients who are nonresponsive to ciclosporin alone or systemic steroids.^13^ Secukinumab, an anti‐IL‐17A monoclonal antibody, is currently the only biologic indicated in children with GPP.^44^, ^45^, ^47^, ^48^, ^49^, ^50^, ^51^, ^52^, ^54^, ^55^

According to Japanese guidelines, systemic inflammatory response syndrome in children should be treated in the same way as acute phase GPP, i.e., with systemic corticosteroids, ciclosporin, etretinate, TNF‐α inhibitors, and granulocyte/monocyte adsorption apheresis.^13^ In rare cases with pulmonary complications related to the underlying disease or those induced by psoriatic medications such as methotrexate in GPP, respiratory management, antibiotics, and discontinuation of the causative drug, with concurrent systemic administration of corticosteroids, are recommended.^13^ GPP may be complicated by arthritis, in which case an effective approach is to treat the arthritis according to the guidelines for treating rheumatoid arthritis, in which case methotrexate or TNF‐α inhibitors are recommended.^13^

Because of the current limitations in available treatments and indications for use, there is an urgent need to develop treatments for pediatric GPP.

## 7 CONCLUSIONS

Pediatric psoriasis has complex symptoms and its treatment requires comprehensive evaluation of the related comorbidities as well as the treatment options and risks. The emotional development of pediatric patients and the effects of psoriasis on the patient's appearance should also be considered. Dermatologists play an essential role in treating children with psoriasis and managing their symptoms, but they must work in collaboration with primary care physicians and other specialists as required.

It is important to educate patients and guardians in the management of pediatric psoriasis. By providing information about the disease, treatment options, and the impact of lifestyle factors (such as increased disease severity in obese individuals), clinicians can enhance the therapeutic partnership, promote shared decision‐making, and improve patient satisfaction and compliance.^9^ Currently, psoriasis treatments rely on experience and expert consensus because of the lack of standardized evidence‐based guidelines, making treatment challenging. There is an urgent need for disease type‐specific evidence‐based guidelines on the treatment of pediatric psoriasis.

Several options for treating psoriasis are currently available, including biologics, topical treatments, and systemic therapies. However, no single treatment is effective in all patients, and different treatments may be needed for different individuals. Cytokines play a significant role in the development of psoriasis, and cytokine expression levels can vary between patients. Researchers have already identified several cytokines that are involved in the disease, such as TNF‐α, IL‐12, IL‐23, IL‐17A, IL‐17F, and IL‐36, and in the future, tailoring targeted therapy according to a patient's individual immunophenotype may significantly improve the treatment of pediatric psoriasis.

## CONFLICT OF INTEREST STATEMENT

A.M. has received grants or contracts from AbbVie GK, Kyowa Kirin Co., Ltd., LEO Pharma K.K., Maruho Co., Ltd., Torii Pharmaceutical Co., Ltd., Taiho Pharmaceutical Co., Ltd., Mitsubishi‐Tanabe Pharma Corp., Sun Pharmaceutical Industries Ltd., and Eisai Co., Ltd.; consulting fees from Nippon Boehringer Ingelheim Co., Ltd., Novartis Pharma K.K., AbbVie GK, Nichi‐Iko Pharmaceutical Co., Ltd., and Nippon Kayaku Co., Ltd.; and payment or honoraria for lectures, presentations, or speakers bureaus from AbbVie GK, Eli Lilly Japan K.K., Eisai Co., Ltd., Janssen Pharmaceutical K.K., Kyowa Kirin Co., Ltd., Maruho Co., Ltd., Mitsubishi‐Tanabe Pharma Corp., Pfizer Japan Inc., Sun Pharmaceutical Industries Ltd., Torii Pharmaceutical Co., Ltd., UCB Japan Co. Ltd., Taiho Pharmaceutical Co., Ltd., and Novartis Pharma K.K. In addition, A.M. has participated on an Advisory Board for Bristol‐Myers Squibb Company, Eli Lilly Japan K.K., and UCB Japan Co. Ltd. H.S. has received grants or contracts from Taiho Pharmaceutical Co., Ltd., Maruho Co., Ltd., Eisai Co., Ltd., Torii Pharmaceutical Co., Ltd., AbbVie GK, LEO Pharma K.K., and Sun Pharma Japan Ltd.; and honoraria for lectures from Mitsubishi‐Tanabe Pharma Corp., Taiho Pharmaceutical Co., Ltd., Torii Pharmaceutical Co., Ltd., Maruho Co., Ltd., Kyowa Kirin Co., Ltd., AbbVie GK, Novartis Pharma K.K., Eli Lilly Japan K.K., LEO Pharma K.K., Celgene Corporation, Janssen Pharmaceutical K.K., Amgen Inc., and UCB Japan Co. Ltd. H.S. is an Editorial Board member of *The Journal of Dermatology* and a co‐author of this article. To minimize bias, he was excluded from all editorial decision‐making related to the acceptance of this article for publication.
